# Supplementary material for: Active layer depth and soil properties impact specific leaf area variation and ecosystem productivity in a boreal forest
Source: PLoS One. 2020 Dec 31;15(12):e0232506. doi: 10.1371/journal.pone.0232506 (PMC7775069; doi:10.1371/journal.pone.0232506)
Supplement: S1 File — (DOCX) [file pone.0232506.s001.docx]

S1 File:

Active layer depth and soil properties impact specific leaf area variation and ecosystem productivity in a boreal forest

Carolyn G. Anderson^1¤^*, Ben Bond-Lamberty^2^, James C. Stegen^1^

^1^Pacific Northwest National Laboratory, Richland, WA, USA

^2^Pacific Northwest National Laboratory, Joint Global Change Research Institute, College Park, MD, USA

^¤^ Current address: Stockbridge School of Agriculture, University of Massachusetts Amherst, Amherst, MA, USA

*Corresponding author, [cganderson@umass.edu](mailto:cganderson@umass.edu)

**Data accessibility**

SLA data used in this study are available in the TRY database (<https://www.try-db.org/>, dataset ID 624). Additionally, SLA, topographic, and edaphic data used in this study can be accessed in the digital data catalog of the Bonanza Creek Long-Term Ecological Research site under project name “Carbon Dynamics Along a Permafrost Gradient at Caribou-Poker Creeks Research Watershed (CPCRW) in Interior Alaska” (<http://www.lter.uaf.edu/>, see full DOI list below). All analysis code is available at <https://github.com/careanderson/cpcrw-sla>. The data underlying this study are owned by the authors.

DOI list:

<http://dx.doi.org/10.6073/pasta/ff37f47d27b4a7d4fba6a09b41d7f964>

<http://dx.doi.org/10.6073/pasta/d768acc8eaa62c9de62601a5c0bc4fb1>

<http://dx.doi.org/10.6073/pasta/047bab8aa2edf323aecdd94ac31a5bc0>

<http://dx.doi.org/10.6073/pasta/3d8077cefdbc5e4ed29cc057aa2c70bd>

<http://dx.doi.org/10.6073/pasta/c1ac609806955be7514f944c5b94786d>

<http://dx.doi.org/10.6073/pasta/07cc9e6ec932d07a481ab3fff24d0376>

<http://dx.doi.org/10.6073/pasta/846d476f4842f4b23838becac60e43c7>

<http://dx.doi.org/10.6073/pasta/2544c2097ef76da3ad1084b8e25c2432>

**S1 Appendix. Methods for partitioning variation of specific leaf area (SLA) within and among trees.**

To partition the SLA variation both within and among individuals in a tree species, we fitted a general linear model to the variance across leaf and tree scales, with leaf nested in tree, and with separate analyses for each species. Specifically, we used the restricted maximum likelihood (REML) method in R function *lme* in the R ‘nlme’ package, version 3.1-125. To partition the variation in SLA between these two scales, we used R function *varcomp* on this model, from R ‘ape’ package, version 3.4:

varcomp.LME.alder <- varcomp(lme(log(SLA) ~ 1, random = ~1|Tree, data=sla_alder, na.action=na.omit),1)

**S1 Fig.** Field site layout. Large gray circles indicate SLA sampling locations. Small black circles indicate soil sampling locations, in a cyclic sampling scheme in north-south and east-west dimensions (3/10 with 4 m grid cell). Two soil sampling locations (indicated by *) are offset from the cyclic design due to previous coring activities at original locations. Due to offsets, soil data were linearly interpolated to match the SLA data. For reference, active layer depth and tree core data were taken along each transect every 2.5 m and 10 m, respectively.

**** **S2 Fig.** Residual plots highlighting non-linearity for linear regressions of SLA vs. gravimetric soil moisture for (a) alder and (b) spruce. The blue line represents a smooth local regression from loess smoothing, and the shade area represents the 95% confidence level interval for predictions.
